# ID 30 - Desafios na Desincorporação de Tecnologias no SUS: análise das demandas e recomendação da Conitec de 2012 a 2023

**DOI:** 10.5327/2237-9622.2025.v34s1.30

**Published:** 2025-11-25

**Authors:** Stéfani Sousa Borges, Francielli Salles Pinheiro, Fernanda d’Athayde Rodrigues

**Keywords:** Avaliação de Tecnologias em Saúde, Sistema Único de Saúde, economia da saúde, análise documental

## Abstract

**Introdução:**

No contexto do Sistema Único de Saúde (SUS), a gestão eficiente dos recursos é fundamental para assegurar a qualidade e a sustentabilidade dos serviços. A Comissão Nacional de Incorporação de Tecnologias no SUS (Conitec), estabelecida em 2011, desempenha um papel central nesse processo, assessorando o Ministério da Saúde (MS) ao avaliar a incorporação, a alteração e a exclusão de tecnologias em saúde. Embora a maior parte das discussões, no contexto da Conitec, estejam centradas na incorporação de novas tecnologias, a exclusão, ou a desincorporação, é igualmente importante, tanto para a segurança do paciente quanto para a sustentabilidade do sistema de saúde. Assim, este estudo tem como objetivo analisar as demandas de exclusão de tecnologias submetidas à Conitec entre 2012 e 2023, analisar as justificativas apresentadas e os atores envolvidos, para identificar oportunidades de aprimoramento do processo de desincorporação e na gestão de recursos no SUS.

**Método:**

Realizou-se uma análise documental, descritiva e retrospectiva das recomendações de exclusão de tecnologias feitas pela Conitec entre 2012 e 2023. Os dados foram extraídos manualmente dos relatórios publicados no site da Conitec e sistematizados em um banco de dados próprio, no Microsoft Excel®. Nos casos em que um relatório abordava múltiplas tecnologias, procedeu-se à individualização delas como demandas distintas. As justificativas para exclusão foram categorizadas e os resultados apresentados em frequências absolutas e relativas.

**Resultados:**

Durante o período analisado, a Conitec recebeu 1.158 demandas, das quais 790 geraram relatórios de recomendação, 24 relativos à exclusão de tecnologias. Desses, 95% foram favoráveis à exclusão, principalmente devido à ausência ou cancelamento de registro (31,43%) e à existência de alternativas terapêuticas mais seguras e eficazes (28,57%). A maioria das exclusões referiu-se a medicamentos (95,9%). Todas as demandas foram originadas de departamentos ou secretarias do MS, sendo a Secretaria de Ciência, Tecnologia e Insumos Estratégicos (SCTIE) a principal demandante. As principais indicações para exclusão foram doença reumatoide de pulmão (17,57%), hepatite C (12,16%), malária (6,76%), doença de Crohn (6,76%) e terapia antirretroviral (8,11%).

**Conclusão:**

O estudo revela que a exclusão de tecnologias no SUS é frequentemente motivada pela necessidade de atualização constante frente a novas evidências científicas e avanços tecnológicos. A maior parte dessas demandas é motivada pela obsolescência tecnológica, o que reflete o compromisso da Conitec com a manutenção de um SUS eficiente e seguro, entretanto, a falta de critérios padronizados e métodos específicos nessas avaliações é uma limitação. No entanto, a participação limitada da sociedade civil e das associações de pacientes sugere que há espaço para fortalecer o engajamento desses grupos no processo de avaliação de tecnologias. Enfim, para garantir a sustentabilidade do SUS, é essencial adotar revisões periódicas de tecnologias já incorporadas, seguindo exemplos de outros países que equilibram a inclusão de novas terapias com desinvestimento em tecnologias menos eficazes, promovendo um uso mais racional dos recursos públicos.

